# On the existence of momentum in professional football

**DOI:** 10.1371/journal.pone.0269604

**Published:** 2022-06-07

**Authors:** Paul J. Roebber, Bryan M. Burlingame, Anthony deWinter

**Affiliations:** University of Wisconsin at Milwaukee, Milwaukee, Wisconsin, United States of America; Texas A&M University, UNITED STATES

## Abstract

Using United States National Football League play-by-play data for the 2002–2012 seasons, we train a neural network to predict win probability, based on measures of the game state. This predictor’s performance is comparable to the point spread at the start of the game and improves thereafter with little bias. We define a measure of success as the change in a team’s win probability over the course of a possession, and show that streaks in this measure are highly unlikely to be random. Further, this finding holds when controlling for the effects of clock management in the fourth quarter of football games, when win probability can increase incrementally for the leading team as the game continues. By defining momentum as an increase in win probability over the course of at least three successive changes in possession, we show some ability to anticipate its emergence, based on game state, using a second neural network. The possibility of using this knowledge for strategic advantage is discussed. We consider these results in the context of examples from National Football League games, including that from Super Bowl LI (Atlanta Falcons versus the New England Patriots), and end with some discussion of future extensions to this work.

## Introduction

Streaks are recognized by both athletes and sports fans, and are considered by them to be the result of an individual or team’s ability to establish and sustain a level of play that is at or near the peak of their abilities (alternatively, streaks of poor play or losses can also occur). Where these streaks are positive, this sustained excellence is often termed “momentum.” A physiological basis for the existence of momentum can be argued in connection with the so-called “winner effect,” in which a win (variously defined) increases the odds of subsequent winning. In other words, in a competitive environment (as in sports), success begets further success and a string of these small wins is perceived as momentum. The specific physiological mechanism for this is the boost in testosterone and reduction in stress-hormone levels following successful encounters, thus rewarding wins and penalizing losses [1, 2]. A counterargument posits that humans often perceive false patterns owing to our evolutionary history, in which a bias towards increased positive detection of lethal threats would more than compensate for the additional false alarms that would necessarily ensue [3–5]. Thus, what we perceive as momentum could simply be a false interpretation of randomness, as in a streak in a series of coin flips.

With valid arguments on both sides, what do the data show? Attempts to empirically establish that streaks in sports occur non-randomly have met with mixed results. The so-called hot hand in basketball may be such a misperception, based on studies of consecutive field goal attempts by players from a professional basketball team, free throw attempts from a different set of professional players, and controlled shooting experiments from individuals on two university teams [6]. This question was later reevaluated with a much larger free throw data set, obtained from five full professional basketball seasons, and it was concluded that there *is* a weak tendency towards non-random streaks in basketball [7]. Those authors speculate that this simply reflects “better and worse periods” of shooting rather than some specific physiological response (as with success breeding success). Thus, despite extensive research on this topic in various sports, conclusive data are rare [8, 9].

These discrepancies may relate in part to inconsistency in the definition of sports momentum: it means different things to different people in different sporting contexts. Here, we will construct and propose a precise definition of momentum in the context of professional football: *the sustained increase in win probability by a single team over the course of at least 2 successive changes in possession* (i.e., on offense and then on defense, or alternatively on defense and then on offense). This definition is, in a sense, consistent with the physiological interpretation of a series of competitive encounters, where the encounter is defined by the possession (itself composed of a series of plays), such that the ultimate objective of winning the game is broken down into a series of small “wins” on both sides of the football. Notably, this is quite different than the hot hand in basketball in which one follows a single player shooting a ball (either from the floor or the free throw line), a process that is much more comparable to a coin flip than that of multiple teammates contributing to an increasing likelihood of a win through all phases of a football game. In that sense, increasing win probability is integrative and perhaps more congruent with fan sensation that the *team as a whole* is performing well. This approach is justified by the structure of professional football games–a team is allowed up to 4 individual plays to gain 10 yards (a first down), and it is often the case that a more risky plays will be used, within that sequence of plays, depending on the game status (e.g., after a 7 yard gain on first down, a team might try a long pass downfield, which is more likely to be incomplete and result in no yards gained, since there are still opportunities remaining to gain the needed 3 yards). By applying the notion of win probability, we determine whether the outcome of the entire possession leads to an improvement in the teams chances of winning the game. Using this definition, we will show that the occurrence of momentum is very likely not random, and that there exist game conditions which increase the likelihood of its emergence.

It should be understood that in defining momentum in this way, we do not claim that we will capture all situations in which a fan may feel a large shift in the tenor of the game has occurred. For example, in the 1995 Army-Navy football game, Navy led 13–7 midway through the fourth quarter but chose a risky play. At fourth-and goal from the one-yard line, they opted to go for the touchdown rather than to attempt a field goal, and the pass attempt fell incomplete. A Navy touchdown at this stage would not have increased their win probability much, but it is arguable that Army, by stopping the drive, gained motivation. In fact, Army subsequently drove 99 yards to win the game 14–13.

In discussing that game, Reed [10] stated: “… the computer cannot discern the momentum shift from Navy to Army as a result of Navy’s snatching a chance of defeat from the jaws of almost certain victory. By taking a blatantly stupid gamble, and losing it, Navy handed Army a totally unexpected reprieve and incalculable psychological uplift in what is always an extremely emotional game.” Although our win probability calculator (see the Methods section) is based on National Football League (NFL) rather than collegiate data, applying it to this game indicates that by scoring a touchdown, Navy would have increased their chance of a win by an additional 3% over that had they scored on a field goal. By failing in that attempt and turning the ball over on downs, however, they lost almost 9% of their chance to win compared to having kicked a field goal (note that Pro Football Reference gives these changes as 3% and 8%, respectively). Thus, although win probability calculations do capture a shift, our definition of momentum is not satisfied since the shift occurred as a result of only one change in possession. Rather, our definition captures instances in which a particular team is sustainably improving its chances over multiple changes in possession.

## Methods

### Football win probability

**Fig 1:**
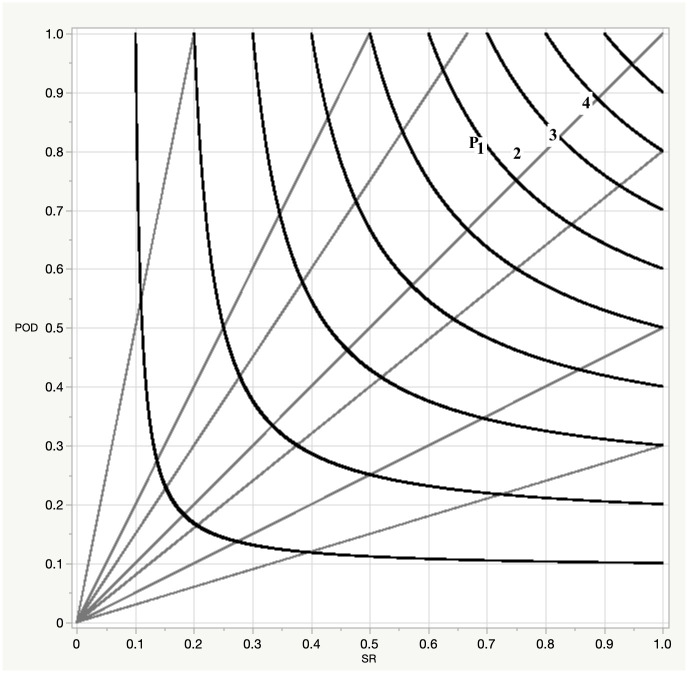
Performance diagram for the artificial neural network (ANN) prediction of win probability. The axes indicate success ratio (SR, equal to one minus the false alarm ratio) and probability of detection (POD). The straight lines emanating from the origin are bias values representing underforecasts below the 45° angle and overforecasts above. The curved lines are the threat score (also known as the critical success index, defined as hits divided by hits plus misses plus false alarms), starting at 0.1 on the lower left and increasing to 0.9 on the upper right. The points shown are performance of the point spread (P), and that of the neural network at the start of the first (1), second (2), third (3) and fourth quarters (4).

Using NFL play-by-play data for the 2002–2012 seasons, it is possible to assign the probability of a win at any point in a game based on conditions at that time. Several such calculators exist online. Here, we establish our own such win probability system, so that we can then apply it to the problem of defining momentum. We train an artificial neural network (hereafter ANN; a multilayer perceptron) with 8 input nodes (yard line, home/visitor possession, quarter, minutes left in quarter, down, first down distance, score differential, and point spread), one hidden layer with 5 nodes, and one output node (probability of a home team win), based upon play-by-play data for 2436 games. Thus, the predictive equation for the win probability (*PrW*) for the game context defined by the above 8 input variables takes the form:

$$PrW=\frac{1}{1+e^{-c+\sum H_{k}}}$$ (1a)

$$H_{k}=\text{tanh}\left[\frac{a_{k}+\sum_{j=1}^{N}b_{jk}V_{j}}{2}\right]$$ (1b)

where *N* = 8 and *k* = 5. We have controlled for overfitting the network by splitting the data into two sections: the first two-thirds for training and the last one-third for validation. Early stopping is then used to halt training, which is done when the performance metric (squared error of the predicted win probability) on the validation dataset no longer improves. We find that the performance of the trained model does not degrade substantially when applied to the validation data (e.g., percent correct, hit rate, critical success index/threat score and bias are all within 5% of that obtained on the training data). Overall, standard performance data [11] for the network are very good (Fig 1).

Some discussion of the performance diagram is provided here. This diagram demonstrates the relationship between POD, FAR, bias, and threat score. Specifically, for a perfect system, one would have POD = 1, FAR = 0, bias = 1, and threat score = 1, a point that would be located in the upper right corner of the diagram. For a given level of skill as measured by the threat score, one can only move along the curved lines denoting the threat score, essentially trading between misses (underforecast bias, indicated by points below the bias = 1 line running at 45° from the lower left to the upper right) and false alarms (overforecast bias, indicated by points above the bias = 1 line). An excellent discussion of verification measures can be found at the World Weather Research Program webpage for forecast verification (https://cawcr.gov.au/projects/verification/).

Not surprisingly, at the beginning of the game, performance mirrors that of the point spread, with probability of detection (POD) and false alarm ratios (FAR) for both around 81% and 32%, respectively. As the game continues, the main improvement with in-game results is a reduction in false alarms and resultant bias, with particularly sharp improvements by the start of the second half, at which time FAR drops to 19%. We note that NFL game conditions have changed over the period from 2002–12; specifically, scoring has increased by 7.5% over this time. Nonetheless, we find that the network performance, which is trained in neglect of these changes, shows no apparent trend through the period, and overall achieves 79% percent correct in its prediction of the winner by the start of the second half. It is possible that incorporating scoring context could improve this performance further, but we note that the probabilities produced by this system are very similar to those produced by the online Pro Football Reference calculator, which suggests that the system we have devised will be suitable for the purposes of this study.

**Fig 2:**
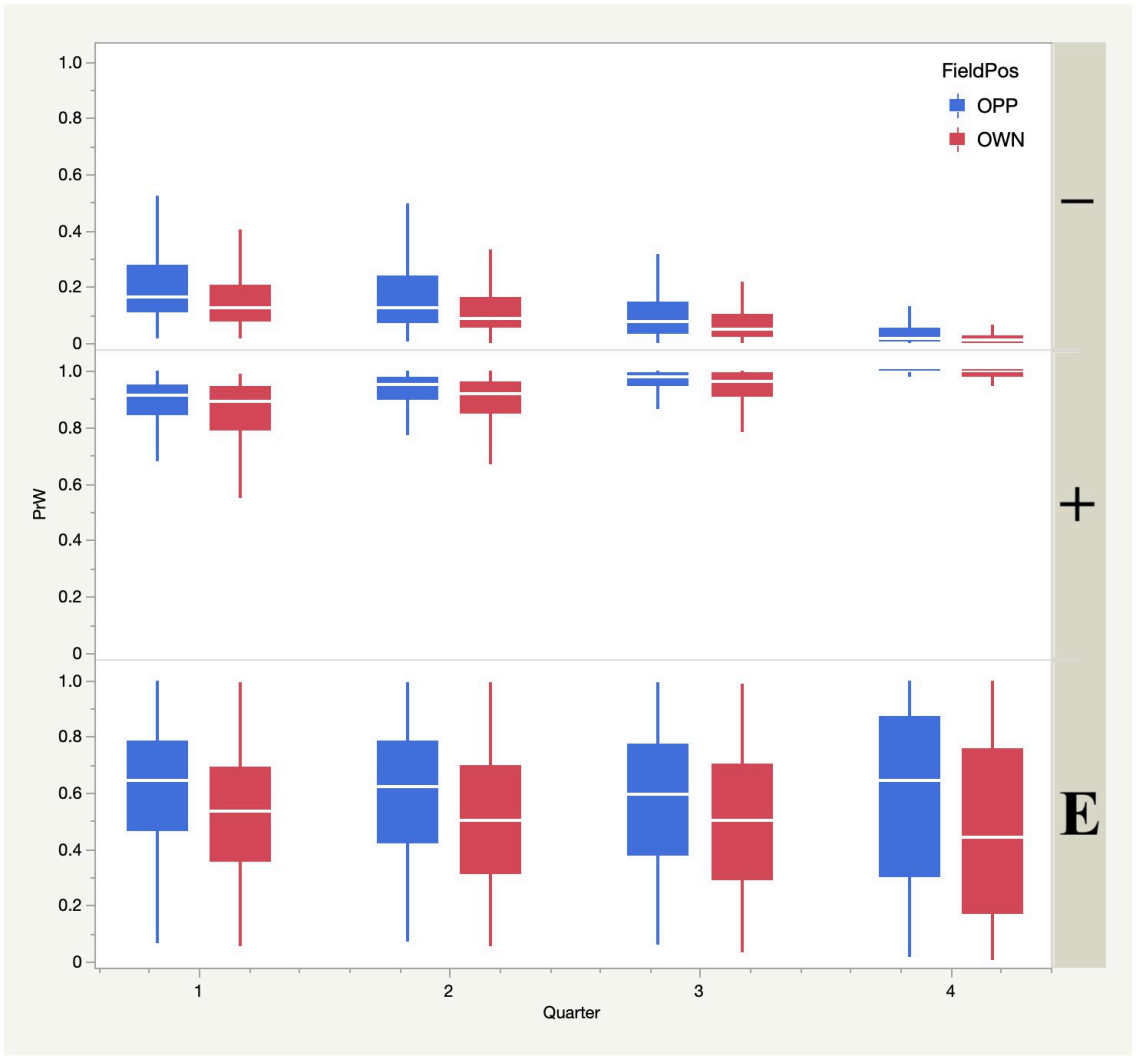
Box plots (10^th^, 25^th^, 50^th^, and 75^th^, and 90^th^ percentile) showing the artificial neural network (ANN) prediction of the probability of a win (*PrW*) as a function of game state. Shown are field position, indicating whether a team has possession on their side of the field (OWN, red) or the opponent’s side (OPP, blue), the quarter, and whether leading (+), trailing (–), or within 1 touchdown (E).

A regression analysis, which can account for the first order effects of the network but cannot capture non-linearities, nonetheless accounts for 84% of the variance in the ANN predictions of win probability (i.e., the R^2^ for this model is 0.84), where the inputs to the regression are (in order of importance, based on logworth): score differential, point spread, yard line, home/visitor possession, quarter, minutes left in quarter, first down distance and down, and all of these inputs have p-values less than 0.001. Using the regression as an interpretive device for the ANN win probability, we find (approximately) 5% is added by home field advantage (compared to 14% in actual won-loss data for this same period), 16% is added per 7 point score advantage, 12% is added per 7 point spread advantage, 1% is added per 10 yards gained, and 2% is subtracted per quarter played. Reflective of the true nonlinearity of the underlying data, the effect of score on win probability is clearly contingent upon game conditions (field position, quarter, score differential; Fig 2), a result consistent with the experience of those with knowledge of the game. For example, in Fig 2, a team that is trailing by more than a touchdown (–) in the first quarter already has a low win probability (median chance of a win less than 20%), but this chance is nearly zero by the fourth quarter. Where a team is within one touchdown, however, field position matters much more, and this is particularly apparent in the fourth quarter, as expected since any lead can be erased or expanded upon with one score.

Super Bowl LI, which featured the Atlanta Falcons versus the New England Patriots, provides an illustrative example of both win probability as computed with the network described above and momentum (see the section on analysis of streaks). In the second quarter of that game, between 14:08 (with a Patriot fumble and loss of possession) and 2:36 (with an interception leading directly to an Atlanta touchdown), the Falcons saw their lead and win probability increase to 21–0 and 92.9%, respectively. As we will show below, this run easily satisfies our definition of momentum. The Falcons win probability peaked at 99.3% at 2:05 in the 3^rd^ quarter with a 28–9 lead and possession of the football at the New England 41 yard line after a failed Patriots onside kick. The game turned beginning at 8:24 in the 4^th^ quarter on the Atlanta 25 yard line, following a Falcon fumble and loss of possession with the Patriots trailing 28–12. At this time, the Atlanta win probability was still very high (98.0%), and as we will show, with limited time remaining in the game, the likelihood that the Patriots would establish momentum (as well as their eventual win) was very small.

### Analysis of streaks

Using the trained ANN defined by (1a) and (1b), we calculate the win probability (*PrW*) for the home team at the beginning of a team’s possession and at the start of the next (opposing team’s) possession. Please note that since we need to measure relative changes we do so relative to the home team, but a *home team increase in PrW is identical to the decrease in PrW for the away team*. *Formally*, *we define*:

*Definition 1*: *The measure is the change in PrW over two successive changes of possession*.
*Definition 2*: *A run is any successive positive or negative values of the measure defined by Definition 1*, *without regard to changes in the quarter or half*.

For example, perhaps the home team has possession and is able to move the ball downfield, but is unable to score. After a punt, the visiting team takes over but is pinned deep in its own territory. Likely, this would lead to an increase in win probability for the home team and would constitute a “win” for that possession (positive measure as in Definition 1). If the home team than held the visiting team to 3 failed attempts for a first down and a punt, leaving the home team with the ball in excellent field position, this would likely lead to a further increase in the home team’s *PrW*, and another possession “win” (i.e. another positive value of the measure). This sequence would constitute a run of length two as per Definition 2. Note that runs of length 1 are allowed, so had the visiting team improved its situation leading to a decrease in PrW for the home team, then we would have a positive measure followed by a negative measure, or two runs each of length 1.

This procedure resulted in 31,572 run instances, with 16,566 streaks of positive increases in win probability. The maximum positive run length was 16 possessions, which occurred on 16 January 2010 in a game between the Baltimore Ravens and Indianapolis Colts. The streak started with a Ravens possession at their own 20 yard line at 11:59 in the second quarter with the scored tied 3–3. The Ravens were forced to punt after three offensive plays, and the Colts moved the ball 75 yards over the next 8 minutes to score a touchdown and extra point and take a 10–3 lead, increasing their win probability to 87%. This probability continued to increase over the remainder of the game, and the Colts won by a score of 20–3, exceeding the point spread by 11.

**Fig 3:**
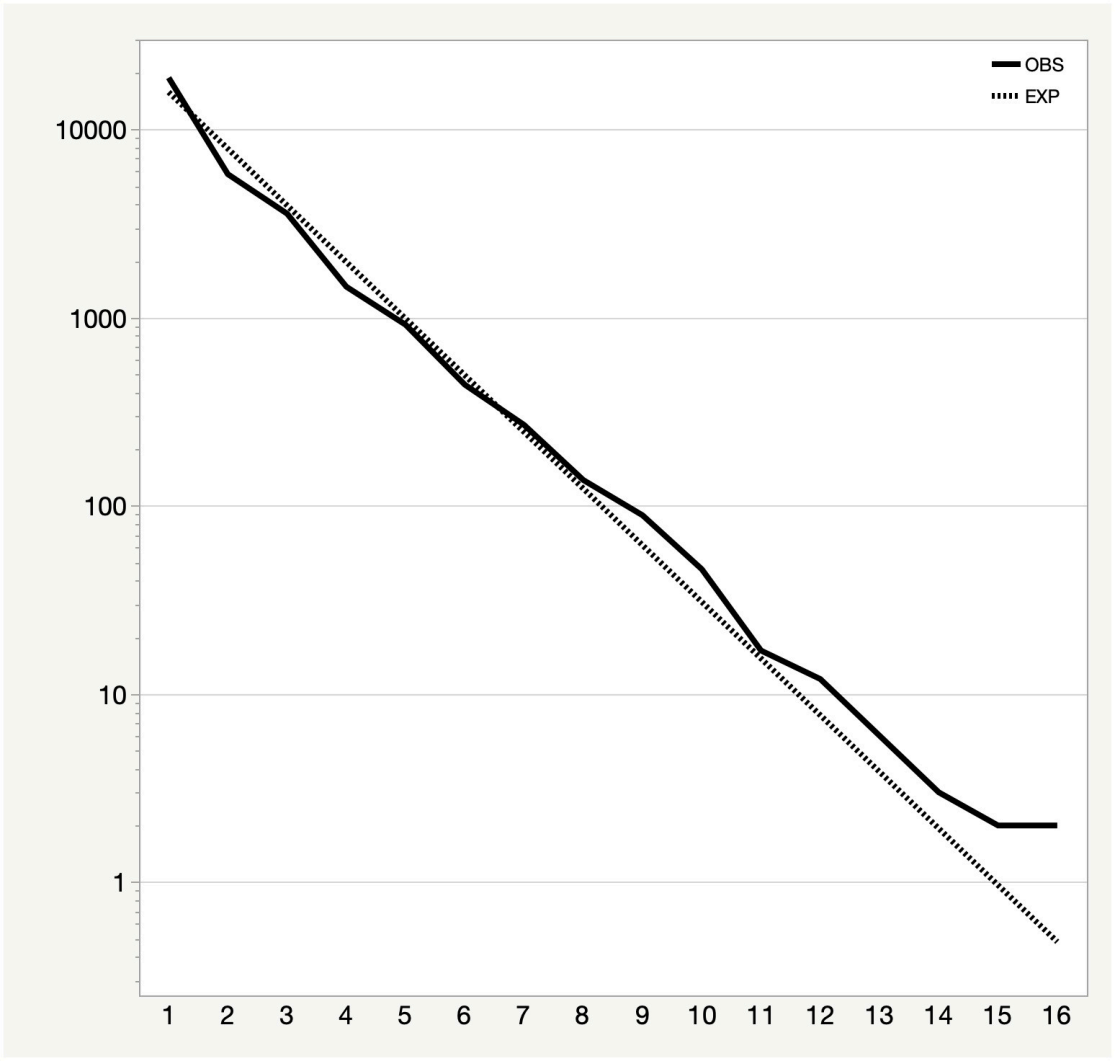
Observed (solid) and expected (dotted) occurrences of runs of a given length (ranging from 1 to 16). Note the logarithmic scale for occurrences.

We apply a length of runs test in which the null hypothesis (H_0_) is that Bernoulli trials (successes, here an increase in win probability, and failures, a decrease in win probability) are independent with a common probability of success. The unconditional probability of a run of length *k* or more possessions is therefore

$$p\left(k\right)=\frac{1}{2^{k}}$$ (2)

and we compute the observed and expected probabilities for run lengths from 1 to 16 [12]. We then use a chi-square test to accept or reject H_0_. We find, based on this test, that we can reject the null hypothesis, with a p-level below 0.0001, that is, these runs are highly unlikely to occur by chance. Notably, it is the long streaks that occur substantially more frequently than would be expected by chance, as indicated by the separation between the lines for observed and expected streaks of lengths 9 to 16 and especially from lengths 12–16 (Fig 3).

There is a confounding factor related to the structure of the game itself that must be considered in understanding this result. Specifically, so-called clock management is an important strategic consideration in how the game is played [10]. Since a football game ends when the game clock runs out, teams with a lead in the fourth quarter can increase their chances of winning simply by running plays that use more time. In our data, we find that a team’s chance of winning increases by 0.4% per minute midway through the fourth quarter with a two score (14 point) lead without any other situational changes. This rate is larger (1% per minute) with a one score (7 point) lead, since the trailing team initially has a chance of making up this difference but as time runs out, their *PrW* diminishes rapidly. Thus, some of the streaks in the fourth quarter may be categorized as a consequence of clock management rather than what many observers would consider as momentum in the usual sense. Further evidence of this limitation is provided by noting that if the change in win probability across the entire dataset (for all quarters) is split into three equally likely ranges (increases of 1.9% or more, decreases of 1.9% or more, and values in between), this middle range occurs considerably more frequently in the fourth quarter (55% of all fourth quarter win probability changes).

In order to test whether fourth quarter streaks can be considered non-random, we use the above three categories and use a 3-category extension of the single-sample runs test [13]. We find that we can reject the null hypothesis that the number of fourth quarter runs is that obtained from a random sample with well above 99.9% confidence. It is interesting to note, however, that the number of observed fourth quarter runs is 16% *below* what one would expect from random chance. Since this is particularly a characteristic of those runs that might be considered momentum (increases of 1.9% or more, indicating improvement in the home team’s chances; decreases of 1.9% or more, indicating improvement in the visiting team’s chances), their relative rarity may be another another reason why observers view such streaks as noteworthy.

The previous example of the game between the Ravens and Colts on 16 January 2010 illustrates the effect of clock management. Recall that this game produced a run of 16 possessions, beginning in the second quarter and continuing through the fourth quarter. When applying the 3-category analysis, this run is reduced to 4 possessions over the second quarter as the Colts increased their win probability by 2.5, 1.9, 2.9 and 7.0% to 94.9% and a 17–3 lead. The remaining 12 possessions in the original run of 16 featured small increases reflecting lead preservation and reducing time on the clock. Thus, the 3-category approach is successful in this example in highlighting the important run of possessions when the Colts took control of that game, and eliminating the “false positive” of clock management in the fourth quarter.

On a related note, it is reasonable to ask whether the occurrence of such streaks results in a greater chance of winning the game. We find, using the 3-category criteria, that winning teams had an average of 1.30 streaks versus 1.14 for losing teams. By applying the Wilcoxon test, we determine that the number of streaks for winning teams is significantly different than that of losing teams, at well above the 99% level of significance.

### Prediction of streaks

Perhaps of equal importance to the result that *win probability streaks in football are non-random*, and that more such streaks are likely to result in wins, is whether or not we can anticipate them. At the beginning of a possession, we categorize the data point as either being attached to the first possession in a series in which momentum subsequently emerges, or not. Here, we will use a more stringent definition of momentum which we consider to be a streak of minimum length three possessions, and for which the *increase* in win probability on each possession is at least 1.9% (in other words, the prediction will be for home team momentum). We include as null cases those instances in which the middle range is indicated, also for streaks of minimum length three in order to provide a more balanced training dataset. For simplicity, we consider only cases where the streak begins with home team possession. This results in a sample of 6755 instances, with approximately 49% of these instances defined as positive momentum. We randomly exclude one-third of the sample for validation.

The neural network discussed previously provides an estimate of *PrW* as a function of game state but does not provide a prediction of the likelihood of positive momentum. Hence, we train a new neural network, but for which N = 4 and k = 7, and the 4 input variables are win probability, time in the quarter, score difference, and total points scored to that point in the game (by both teams) and the desired output is the probability of positive momentum. The performance of this network on the validation data is quite good, with POD and FAR of 62% and 36%, respectively, for an overall threat score of 0.458. Thus, there is an ability to detect when the emergence of momentum is more likely, with a slight tendency to underpredict (bias equals 0.975). For the Ravens-Colts run discussed above, the predictor indicates a 55% probability of occurrence of such a longer streak.

**Fig 4:**
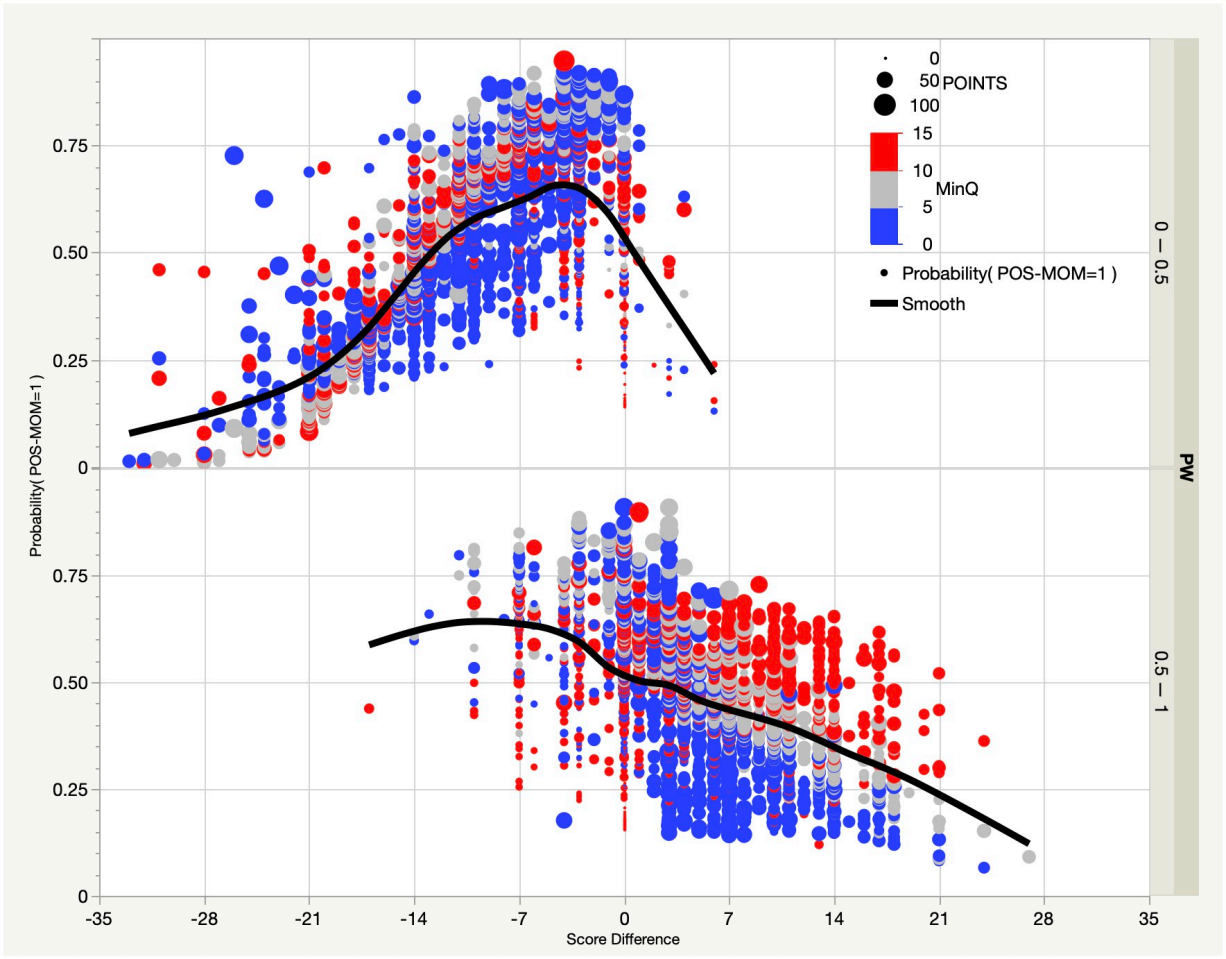
Artificial neural network (ANN) predictions of probability of momentum, defined as a streak of minimum length three increases in home team win probability, beginning with possession of the ball by the home team, where all inputs are at the start of the streak. Shown are the score difference (relative to the home team, where negative indicates the home team trailing), minutes remaining in the quarter (colors, where red, grey and blue are 10–15 minutes, 5–10 minutes and less than 5 minutes, respectively), points scored by both teams by that point in the game (circle sizes), for conditions in which the home team win probability is less than (top) or greater than (bottom) 50%. The black lines show smoothed fits to the data.

We also find that the momentum prediction is highly nonlinear (Fig 4), with only 2.3% of the probability accounted for by a multiple linear regression fit to the ANN using the 4 input variables. There is some tendency for momentum to develop earlier in the quarter (red circles) and when there has been less overall scoring (small circles). Momentum by the home team is more likely to develop when the score difference is within a touchdown but particularly when the home team is trailing (e.g., score difference equal to -7) and their win probability is less than 50% (top half of Fig 4). Interestingly, this finding suggests the idea of a comeback motivation. At first glance this might seem to run counter to the suggestion of the physiological research regarding the winner effect, but it should be noted that we are making the prediction *at the start of the run*, whereas the run itself is what is defined by the series of wins.

Consider again Super Bowl LI (Atlanta Falcons versus the New England Patriots, played on February 5, 2017; note the Roman Numeral notation is a conceit of the National Football League owners, a practice that was begun with the 5^th^ Super Bowl held, on January 17, 1971). In the second quarter between 14:08 and 2:36, the Atlanta Falcons saw their win probability increase in consecutive changes of possession in increments of 12%, 15%, 11%, and 14%. This streak, which easily satisfies the more strict definition of momentum discussed above, began with a Patriots fumble and loss of possession at the Atlanta 29 yard line. At this point however, the chance of establishing momentum was only 21%, as there had not yet been any scoring in the game.

In the third quarter with a 21–3 lead, the Falcons put together another, shorter streak. It began after a Patriots failed to get a first down on three plays, and punted to give the Falcons the ball at their own 15 yard line at 12:45. At 8:31, Bryant kicked the extra point following a Falcon 85 yard touchdown drive, making the score 28–3 Atlanta and increasing their win probability by 6.7% to 98.4%. The Patriots then drove the ball 75 yards to score, but also took more than 6 minutes off the clock, missed the extra point, and failed to recover their onside kick. This made the score 28–9 Atlanta and the Falcon’s ball at the New England 41 yard line with 2:05 left in the third quarter, increasing Atlanta’s win probability to 99.3%. Had Atlanta scored a touchdown on this possession, they would have further increased their win probability to 99.8% and extended their streak, based on the less strict definition. Instead, they were forced to punt, giving New England the ball on their own 13 yard line with 14:51 left to play, slightly lowering Atlanta’s win probability to 98.5%. Although this streak does not qualify according to our more strict criteria, many would consider this sequence to be a noteworthy series in the game.

When the Patriots took the ball at 8:24 in the fourth quarter on the Atlanta 25 yard line after a Falcon fumble, the game began to turn. Trailing by 16 points at this point, however, the Patriots had only a 2% chance of winning. The Patriots scored a touchdown and made a two point conversion, narrowing the gap to 8 points, but with only 5:53 left to play, the Falcons still had a 91% chance of winning and most importantly, had possession of the ball. This latter is owing to the aforementioned clock management effect, where win probability increases for a team with a lead simply by their taking time off the clock. Although the Falcons failed to score, by using another 2.5 minutes, they increased their chance of a win to 96%. Remarkably, the Patriots then drove the ball 91 yards to score and made another two point conversion, tying the game and flipping the win probability to 60% in their favor with less than a minute to play in regulation. This extraordinary sequence of events also does not constitute momentum based on our strict definition, since the Patriot win probability changes from 8:24 were 6.7%, -4.7%, and 56.1% (our predictor indicates the chance of establishing momentum was low but not entirely improbable at 35%). We argue that a better categorization of this sequence might be as a “Black Swan event” rather than momentum as it is generally understood.

## Discussion

Given that momentum, as defined in this paper, exists and that there is some means of anticipating its development, the question arises as to whether this determination can lead to any strategic advantage during game play. The data suggest that it may be possible in some circumstances to “prime the pump,” that is, to increase the likelihood of establishing momentum.

As discussed previously, (home team) momentum is most likely to develop when the score is relatively close and there is time on the clock. A defensive stop by the home team, followed by a punt that leaves their offense in relatively good field position would increase their win probability. If the offense scored, this could begin the process of momentum, following the old adage of “building on success.” Ultimately, such a scenario requires execution by the defense and perhaps also special teams, but it provides a stronger rationale for a focus on the performance of all parts of a team.

Beyond these aspects, in the fourth quarter, there is leverage available based on time on the clock (previously noted as approximately 1% per elapsed minute in a close game). Here, the importance of defense becomes more evident. Consider a hypothetical scenario in which two evenly matched teams are tied at 21 midway through the 4^th^ quarter, and the visitors have the ball on their own 25 yard line. The home team win probability is 41%. Suppose their defense holds the opposition to 3 offensive plays and a punt, and in doing so only 1 minute has elapsed, while they gain possession of the ball on their own 25 yard line. The defense has increased their win probability to 49%. If they then use 3 minutes to score a field goal, their win probability increases to 70%. If their defense holds again, and another minute comes off the clock, their win probability has increased further to 83%. This sequence of possessions, two involving the defense, has established momentum by our strict standard. If the offense follows this with another score and more time off the clock, the streak is extended.

We note that from the point when the home team has possession with a win probability of 49%, our predictor indicates an 81% chance that momentum will develop given that game state–here we are excluding the first possession in that sequence since we have defined our momentum predictor relative to the home team having possession of the ball, but the streak actually begins with the first defensive sequence.

## Conclusions and possible extensions

In prior studies, it has been difficult to establish the existence of non-randomness in sports streaks (e.g., the “hot hand” in basketball). Here, we have defined streaks in a way that is both consistent with athlete and fan perception of sustained, *high-level team performance* and with physiological research that may pertain to such occurrences. Using National Football League play-by-play data, we have established that streaks formed by increases in team win probability over successive possessions of minimum length two are highly non-random and are a function of the game state, suggesting some ability to increase the chances of its occurrence through strategic adjustments. We find this non-randomness persists even when we control for intrinsic characteristics of the game, such as time on the clock, which can produce long streaks in the fourth quarter simply by the leading team controlling the ball. Future research directions could include:

1. Further exploration of strategies, perhaps at the level of the calling of individual plays, to determine whether play sequencing leads to greater or lesser chances of establishing team momentum.
2. Time-dependent forms of the model(s), which might be able to account for changes in the style of play in the NFL (i.e. scoring has increased by 7.5% over the period of the present study) and provide further improvements to the present estimations.
3. Undertaking similar studies in other team-based sports, such as the National Basketball Association (NBA) games, where play-by-play data might provide evidence of non-random streakiness, previously not yet identified.

## Supporting information

S1 File (CSV)

S2 File (CSV)
